# Endothelium-related biomarkers enhanced prediction of kidney support therapy in critically ill patients with non-oliguric acute kidney injury

**DOI:** 10.1038/s41598-024-54926-9

**Published:** 2024-02-21

**Authors:** Francisco Thiago Santos Salmito, Sandra Mara Brasileira Mota, Francisco Márcio Tavares Holanda, Leticia Libório Santos, Luana Silveira de Andrade, Gdayllon Cavalcante Meneses, Nicole Coelho Lopes, Leticia Machado de Araújo, Alice Maria Costa Martins, Alexandre Braga Libório

**Affiliations:** 1 Postgraduate Program, Rede Nordeste de Biotecnologia - RENORBIO, Fortaleza, Ceará Brazil; 2 Centro de Informação e Assistência Toxicológica do Instituto José Frota – IJF, Fortaleza, Ceará Brazil; 3 Medical Program, Universidade de Fortaleza- UNIFOR, Fortaleza, Ceará Brazil; 4 Medical Sciences Postgraduate Program, Department of Internal Medicine, Medical School, Federal University of Ceará, Fortaleza, Brazil; 5 Pharmacology Postgraduate Program, Department of Physiology and Pharmacology, Medical School, Federal University of Ceará, Fortaleza, Brazil; 6 Clinical and Toxicological Analysis Department, School of Pharmacy, Federal University of Ceará, Fortaleza, Brazil; 7 Medical Sciences Postgraduate Program, Universidade de Fortaleza- UNIFOR, Fortaleza, Ceará Brazil; 8 Medical Course, Universidade de Fortaleza-UNIFOR, Fortaleza, Ceará Brazil

**Keywords:** Acute kidney injury, Kidney support therapy, Endothelium-related biomarkers, Syndecan-1, Prognostic markers, Translational research

## Abstract

Acute kidney injury (AKI) is a common condition in hospitalized patients who often requires kidney support therapy (KST). However, predicting the need for KST in critically ill patients remains challenging. This study aimed to analyze endothelium-related biomarkers as predictors of KST need in critically ill patients with stage 2 AKI. A prospective observational study was conducted on 127 adult ICU patients with stage 2 AKI by serum creatinine only. Endothelium-related biomarkers, including vascular cell adhesion protein-1 (VCAM-1), angiopoietin (AGPT) 1 and 2, and syndecan-1, were measured. Clinical parameters and outcomes were recorded. Logistic regression models, receiver operating characteristic (ROC) curves, continuous net reclassification improvement (NRI) and integrated discrimination improvement (IDI) were used for analysis. Among the patients, 22 (17.2%) required KST within 72 h. AGPT2 and syndecan-1 levels were significantly greater in patients who progressed to the KST. Multivariate analysis revealed that AGPT2 and syndecan-1 were independently associated with the need for KST. The area under the ROC curve (AUC-ROC) for AGPT2 and syndecan-1 performed better than did the constructed clinical model in predicting KST. The combination of AGPT2 and syndecan-1 improved the discrimination capacity of predicting KST beyond that of the clinical model alone. Additionally, this combination improved the classification accuracy of the NRI and IDI. AGPT2 and syndecan-1 demonstrated predictive value for the need for KST in critically ill patients with stage 2 AKI. The combination of AGPT2 and syndecan-1 alone enhanced the predictive capacity of predicting KST beyond clinical variables alone. These findings may contribute to the early identification of patients who will benefit from KST and aid in the management of AKI in critically ill patients.

## Introduction

Acute kidney injury (AKI) is a common condition in hospitalized patients and can be caused by a variety of factors, including sepsis, hypotension, nephrotoxic drugs, and other medical conditions^1^. In addition to causing high mortality, AKI is also associated with bleeding, coronary heart disease, stroke and chronic kidney disease (CKD)^2^. The incidence of AKI is highest in critically ill and septic patients, with an increasing trend in the use of dialysis in critically ill patients^3^.

In the last two decades, AKI has been classified according to its severity, and with progression, several life-threatening complications can develop, e.g., hyperkalemia, metabolic acidosis, uremic complications, and fluid overload^4,5^. When one or more of these complications develop, kidney support therapy (KST) is generally indicated.

Recently, several large trials have evaluated whether early KST, before any life-threatening complications develop, mainly based on AKI stage, have any impact on survival, KST dependence or CKD progression^6–10^. One common feature of almost all trials is that many patients who underwent late KST initiation had ameliorated renal function and never needed KST. The findings suggest that deferring KST whenever possible may lead to a substantial reduction in the percentage of patients who will ultimately receive KST. However, there is a need to better understand why these patients could avoid KST^11^. The recent ADQI recommendations on AKI biomarkers highlighted the need for further research on the assessment of kidney damage and biomarkers correlated with clinical data to define the optimal timing of KST^12^. Machine learning techniques and the furosemide stress test (FST) have been used to evaluate renal reserve and predict AKI progression and the need for KST^13–17^. Of these, FST is the most promising, demonstrating a good discrimination to KST need outperformed common AKI biomarkers.

In the present study, we aimed to analyze endothelium-related biomarkers collected from critically ill patients with stage 2 AKI defined by serum creatinine levels and their capacity to predict KST need in the next 72 h. For this purpose, we evaluated vascular cell adhesion protein-1 (VCAM-1), which is related to endothelial cell activation; angiopoietin (AGPT) 1 and 2, which interact with and antagonize AGPT1 via the same receptor, Tie2, with equal affinity, whereas AGPT1 induces maturation and stabilization of the endothelium; AGT2 causes destabilization and increases vascular permeability; and, ultimately, syndecan-1, a newly explored marker of endothelial glycocalyx derangement^18^.

## Methodology

### Patient selection

This prospective observational study was performed at a hospital unit that serves as a trauma reference and pursues five adult ICUs with a total of 40 beds. All patients aged 18 years or older admitted to the ICU between November 2021 and November 2022 were screened for the development of stage 2 AKI as defined by the serum creatinine (sCr) criterion only. Baseline sCr levels were defined as the creatinine level upon hospital admission. Patients with medical support limitations, preexisting chronic kidney disease under maintenance hemodialysis, baseline sCR greater than 2 mg/dL, metabolic complications at enrollment (defined as serum potassium > 6 mEq/L, serum urea > 200 mg/dL and metabolic acidosis with pH < 7.2 and serum bicarbonate < 15 meq/L) or with reduced urine output (less than 0.5 mL/kg/h for at least 12 h) were excluded from the study. The exclusion criterion for patients with reduced urine output is justified by the routine use of an FST as a parameter for KST initiation in patients with reduced urine output at our institution. The FST is a common method used in institutions for evaluating renal function, and if the test is negative (urine output less than 200 ml in 2 h)^14^, the KST is generally initiated. This study protocol was reviewed and approved by the Ethical Committee of Hospital Geral de Fortaleza, approval number 1.032.914, and all participants signed free and informed consent before inclusion. The research was conducted ethically in accordance with the World Medical Association Declaration of Helsinki.

### Clinical parameters

Demographic data (age, sex, liver disease) were obtained from direct observation and medical records. Body weight was estimated from height. Patients were screened for the use of vasopressor drugs and mechanical ventilation. On day AKI stage 2 was achieved, 5 ml of blood was collected from each patient for measurement of endothelial biomarkers, as described below. Additionally, the serum potassium concentration, bicarbonate concentration, blood glucose concentration, 24-h urine output, serum urea concentration, previous 24-h sCr increase, cumulative fluid balance during the ICU stay, vital signs, vasopressor use and nonrenal SOFA score were recorded. These variables were collected because they are cited in the literature as predictors of KST in critically ill patients or could influence assistant physicians in the decision to initiate KST^13,15–17^.

### Biomarker measurement

Syndecan-1 was measured as a biomarker of endothelial glycocalyx injury (Abcam, Cambridge, MA, USA). The intra-assay coefficient of variation was 6.2%. Additionally, VCAM-1 was measured using a commercially available ELISA kit (Abcam, Cambridge, UK), with an intra-assay coefficient of 5.9%. AGPT1 and AGPT2 were measured using an ELISA kit (R&D Systems, Minneapolis, MN, USA). The intra-assay coefficients of variation were 4.7 and 5.3%, respectively. All the measurements were performed in duplicate.

### Outcomes

The main outcome was KST initiation up to 72 h after inclusion in the study. The secondary outcomes were the need for KST up to 96 h after study inclusion and ICU mortality. The KST was initiated by an attending nephrologist, but the institution used some instructions to initiate the KST, as described above (for hyperkalemia, metabolic acidosis, and clinical perception of hypervolemia/inadequate urine output to maintain adequate fluid balance, a negative FST and a serum urea concentration > 200 mg/dL). The main reasons for KST initiation were collected.

### Statistical analysis

Continuous variables are described as medians (interquartile ranges) or means and standard deviations, as appropriate, and categorical variables are described as proportions. Continuous variables were compared using a 2-sample t test or Mann–Whitney test, and dichotomous variables were compared with the chi-square or Fisher exact test. We collected important covariates that predict the need for KRT in other studies, namely, age, liver disease status, baseline sCr concentration, change in sCr concentration in the 24 h previous study, 24 h urine output, nonrenal SOFA score, blood glucose, mechanical ventilation, cumulative fluid balance and need for vasoactive drugs. To evaluate the association between biomarkers and the need for KRT in the next 72 h, logistic regression models were used to adjust for those variables that were significant according to univariate analysis. The collected variables were also used to construct a clinical model to predict KST in the next 72 h. To avoid overfitting of the model, we used penalized maximum likelihood estimation, which yielded decreased regression coefficients. The optimum penalty factor that maximized the modified Akaike information criterion was used.

The area under the curve (AUC) for receiver operating characteristic (ROC) curves was calculated for the biomarkers and for the clinical model. After that, endothelium-related biomarkers were added to the clinical model, and the AUC-ROC values were compared using the method of DeLong and colleagues^19^. Furthermore, we calculated the continuous net reclassification improvement (NRI) and integrated discrimination improvement (IDI) for endothelium-related biomarkers regarding the need for KST in the next 72 h to evaluate its additional predictive value. The NRI is described as the percentage of patients whose stratification improved after the addition of the biomarkers under assessment. The risk was determined by calculating the sum of differences in the proportions of patients who moved up minus the proportion who moved down for patients who developed the event and the proportion of patients who moved down minus the proportion who moved up for patients who did not develop the event. The NRI and IDI are recommended as sensitive tools for detecting the additional benefit of a predictive marker. The sensitivity of these methods exceeds changes in areas under receiver operating characteristic curves^20,21^. The IDI is calculated according to the same principle as the NRI, using changes in the model-based probabilities^20^. The calculation of the NRI and IDI was performed using the clinical model as a reference. Decision curve analysis (DCA) was used to verify and evaluate the clinical practicability of the clinical model with and without biomarkers. Analysis of the data was performed using SPSS 20.0 for Windows (SPSS, Inc., Chicago, IL) and R version 2.14.1 (R Development Core Team, Vienna, Austria).

### Ethical approval

This study protocol was reviewed and approved by the Ethics Committee of Hospital Geral de Fortaleza (approval number 1.032.914), and all participants signed free and informed consent before inclusion. The research was conducted ethically in accordance with the World Medical Association Declaration of Helsinki.

## Results

### Characteristics of the study cohort

**Figure 1:**
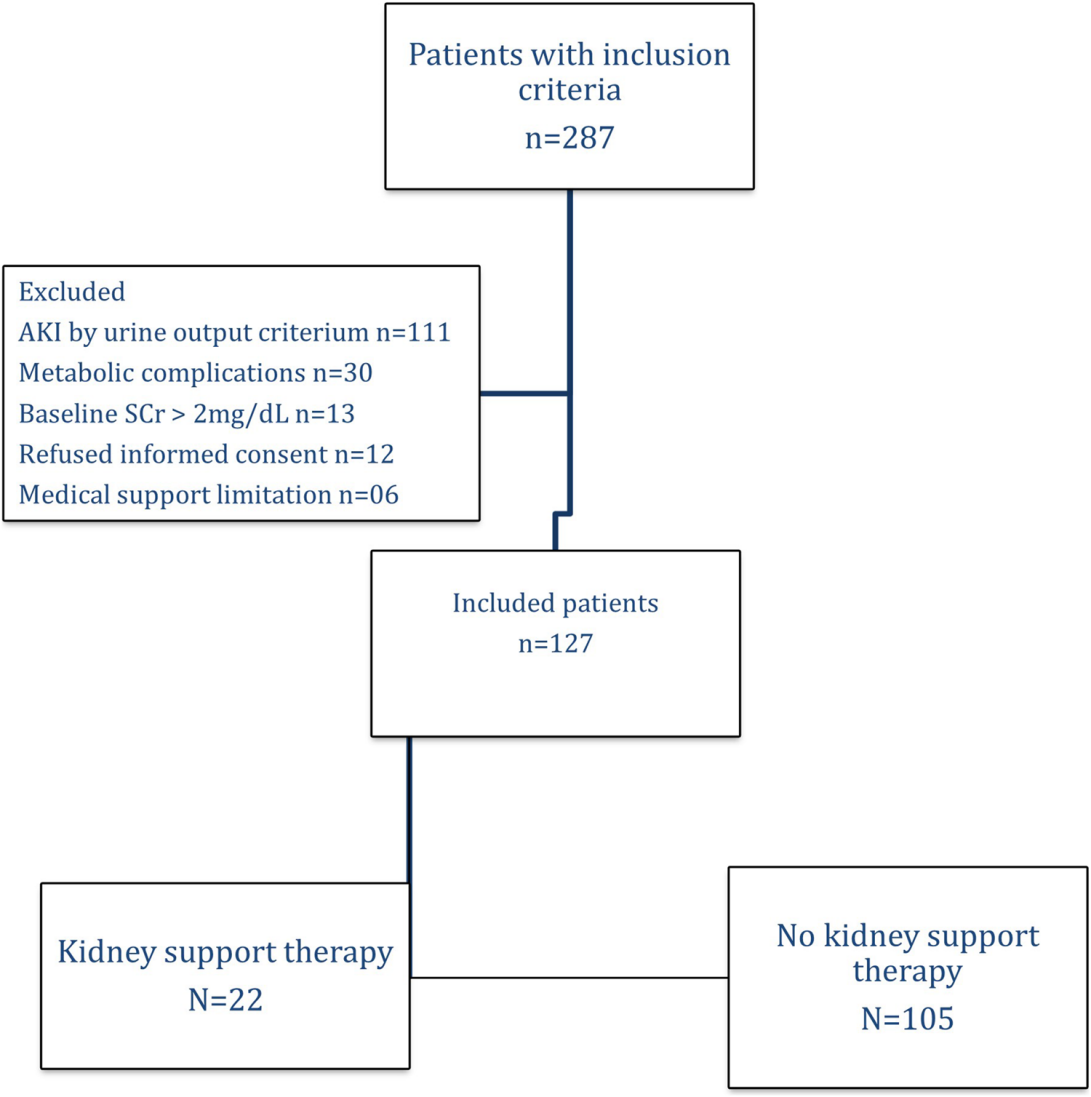
Flowchart of patients and exclusion criteria.

During the study period, 299 patients achieved stage 2 AKI according to the sCr criterion and were considered for inclusion; 172 patients were excluded for the following reasons: AKI stage 2 according to both the urine output and sCr criteria (n = 111), metabolic complications (n = 30), baseline sCr levels greater than 2 mg/dL (n = 13), refusal to provide informed consent (n = 12) and medical support (n = 6). Overall, 127 patients (72.4% males) were included in the final analysis, with a mean age of 42.7 ± 10.2 years (Fig. 1). Although patients were screened daily, 16 (12.6%) were already included in AKI stage 3. The median urine output 24 h after inclusion was 0.7 [0.6–0.8] mL/kg/h. The metabolic variables associated with KST indication are listed in Table 1. Overall, 22 (17.2%) patients needed KST in the first 72 h after study inclusion (Fig. 1), and the hospital mortality rate was 46.5%. Patients who progressed to KRT had a greater SCr increase in the last 24 h before study inclusion (0.9 [0.7–1.4] vs. 0.6 [0.4–0.8] mg/dL, *p* = 0.001) contributing to a higher SCr level at study inclusion 2.2 [1.5–3.2] versus 1.5 [1.4–1.8] mg/dL, *p* < 0.001. The complete patient characteristics according to AKI progression to the KST are displayed in Table 1, and the main reasons for KST are shown in Supplementary Table S1.

**Table 1:** Cohort description of patients according to kidney support therapy initiation 72 h after study inclusion.

	All Patients (n = 127)	Without kidney support therapy (n = 105)	With kidney support therapy (n = 22)	*P*
Age (years), mean ± SD	42.7 ± 10.2	42.1 ± 10.1	45.6 ± 10.5	0.17
Male, *n* (%)	92(72.4)	75 (71.4)	17 (77.3)	0.58
Comorbidities				
Hypertension, n(%)	13 (10.2)	9 (8.6)	4 (18.2)	0.18
Diabetes Mellitus, n(%)	8 (6.3)	7 (6.7)	1 (4.5)	0.71
Congestive heart failure, *n* (%)	5 (3.9)	3 (2.8)	2 (9.1)	0.68
Liver disease, n(%)	5 (3.9)	5 (4.8)	0 (0.0)	0.30
Main diagnosis at ICU admission				
Sepsis, n (%)	67 (52.8)	54 (51.4)	13 (59.1)	0.51
Acute respiratory failure, n (%)	18 (14.2)	13 (12.4)	5 (22.7)	0.21
Hemorrhagic shock, n (%)	15 (11.8)	14 (13.3)	1 (4.5)	0.25
Coma, n (%)	6 (4.7)	6 (5.7)	0 (0.0)	0.25
Other, n (%)	21 (16.5)	18 (17.1)	3 (13.6)	0.69
Nonrenal SOFA score, median [IQR]	5 [2–10]	5 [2–9]	5.5 [3–11]	0.31
Mechanical ventilation, n(%)	92 (72.5)	76 (72.4)	16 (72.7)	0.97
Vasoactive drugs, n(%)	62 (48.8)	47 (44.8)	15 (68.2)	0.04
Baseline SCr (mg/dL), median [IQR]	0.8 [0.6–1.0]	0.8 [0.6–1.0]	0.8 [0.6–0.9]	0.45
Variables on study inclusion				
AKI stage 3 by SCr criterium, n(%)	16 (12.6)	11 (9.2)	5 (22.7)	0.15
Blood glucose (mg/dL), mean ± SD	111 ± 15	111 ± 14	110 ± 15	0.76
SCr (mg/dL), median [IQR]	1.5 [1.3–1.9]	1.5 [1.3–1.8]	2.2 [1.5–3.2]	< 0.001
Serum urea (mg/dL), median [IQR]	69 [56–89]	69 [56–89]	70 [58–100]	0.50
Serum potassium (mEq/L), median [IQR}	4.7 [3.5 –5.4]	4.7 [3.6–5.4]	4.5 [3.4–5.6]	0.65
Serum bicarbonate (mEq/L), median [IQR]	27 [23–32]	27 [24–32]	25 [22–33]	0.26
24 h-urine output (mL/kg/h), median [IQR]	0.7 [0.6–0.8]	0.7 [0.6–0.8]	0.6 [0.5–0.7]	0.63
Cumulative fluid balance (% of estimated BW), median [IQR]	2.2 [0.8–3.1]	2.2 [1.0–3.1]	2.3 [0.8–3.8]	0.27
Change in SCr in last 24 h (mg/dL), median [IQR}	0.6 [0.5–0.9]	0.6 [0.4–0.8]	0.9 [0.7–1.4]	0.001
Syndecan-1 (ng/mL), median [IQR]	489 [225–679]	418 [196–614]	803 [575–980]	< 0.001
VCAM-1 (ng/mL), median [IQR]	1495 [1057–1862]	1386 [1047–1913]	1564 [1218–1787]	0.23
Angiopoietin-1 (mg/L), median [IQR]	3.6 [0.9–5.9]	3.3 [0.9–5.7]	5.8 [1.6–15.0]	0.24
Angiopoietin-2 (mg/L), median [IQR]	3.0 [1.8–5.4]	2.6 [2.6–4.4]	5.6 [2.9–12.3]	< 0.001
Angiopoietin2/1 ratio, median [IQR]	0.9 [0.5–2.3]	0.9 [0.4–2.4]	1.0 [0.8–1.9]	0.23
Hospital mortality, n(%)	59 (46.5)	44 (41.9)	15 (68.2)	0.03

### Association of endothelium-related biomarkers and AKI progression to KRT

No difference in the levels of VCAM or AGPT1 or in the AGPT2/1 ratio was detected between patients who progressed or did not progress to the KST within the next 72 h. However, patients progressing to KST had higher levels of AGPT2—5.6 [2.9–12.3] versus 2.6 [2.6–4.4] mg/L—and syndecan-1—803 [575–980] versus 418 [196–614] ng/mL, *p* < 0.001 for both. No significant association was observed for VCAM-1.

According to our univariate analysis, both AGPT2 (odds ratio (OR) 1.28 [95% confidence interval (OR) 1.13–1.44] for each 1 mg/L) and syndecan-1 (OR 1.72 [95% CI 1.34–2.20] for each 100 ng/mL) were associated with the need for KST 72 h after study inclusion. After we adjusted for clinical and laboratory variables, AGPT2 and syndecan-1 remained independently associated with the need for KST. Additionally, a parsimonious clinical model with basal sCr, a change in sCr in the last 24 h before study inclusion and the use of vasoactive drugs was associated with KST even after adjustment for AGPT 2 and syndecan-1. Univariate and adjusted ORs are shown in Table 2, and forest plots are shown in Supplementary Fig. S1.

**Table 2:** Endothelial-related biomarker levels associated with kidney support therapy initiation 72 h after study inclusion.

	Univariate analysis OR (95%CI)	*P*	Adjusted OR (95%CI)	*P*
Syndecan-1, for each 100 ng/mL	1.72 (1.34–2.20)	< 0.001	2.17 (1.49–3.15)	< 0.001
VCAM-1, for each 1000 ng/mL	1.14 (0.85–1.51)	0.41	0.93 (0.55–1.57)	0.78
Angiopietin-1, for each 1 mg/L	0.93 (0.67–1.15)	0.65	0.91 (0.72–1.27)	0.46
Angiopoietin-2, for each 1 mg/L	1.39 (1.21–1.59)	< 0.001	1.35 (1.16–1.57)	< 0.001
Angiopoietin2/1 ratio, for each 1 unit	1.08 (0.81–1.26)	0.36	1.05 (0.92–1.18)	0.48

Adjusted for baseline sCr, change in sCr after the 24-h previous study and need for vasoactive drugs.

*VCAM* vascular cell adhesion protein.

### Diagnostic testing

**Figure 2:**
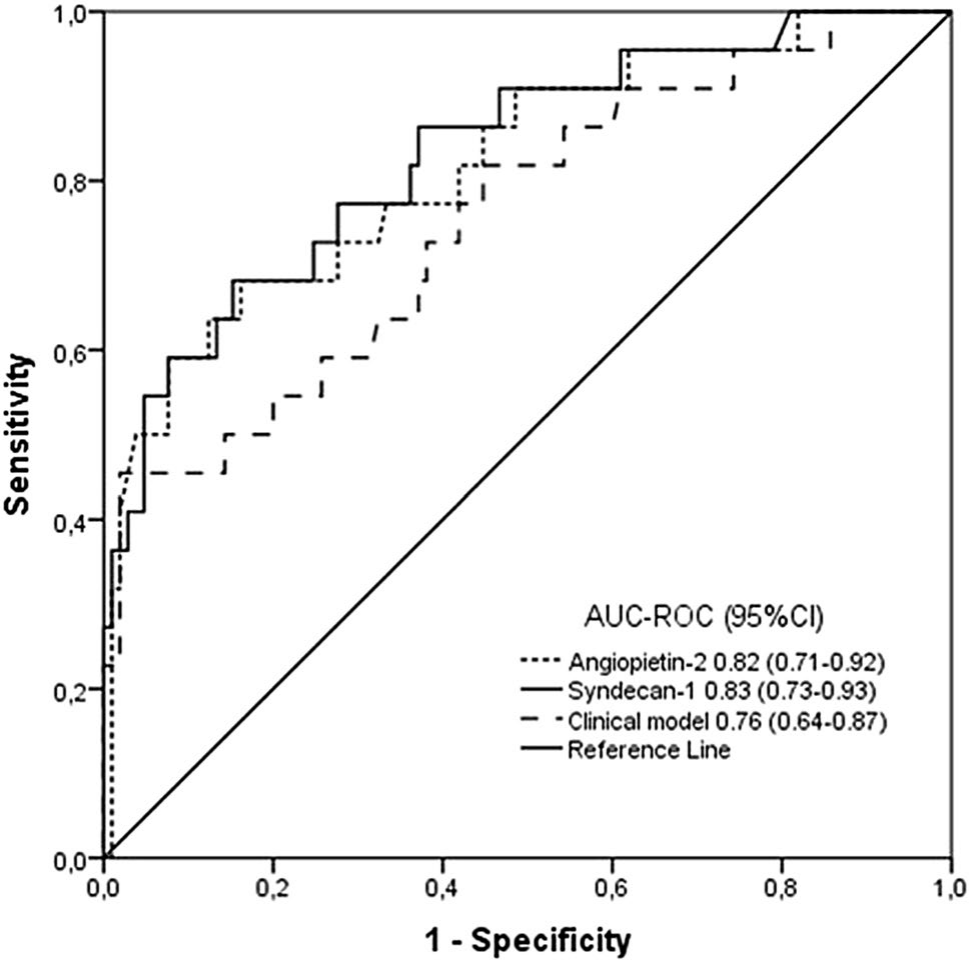
Diagnostic performance of angiopoietin-2, sydecan-1 and a clinical model including serum creatinine in the last 24 h before study inclusion and the use of vasoactive drugs for kidney support therapy in the next 72 h. *p* value < 0.001 for all AUC-ROC curves.

**Figure 3:**
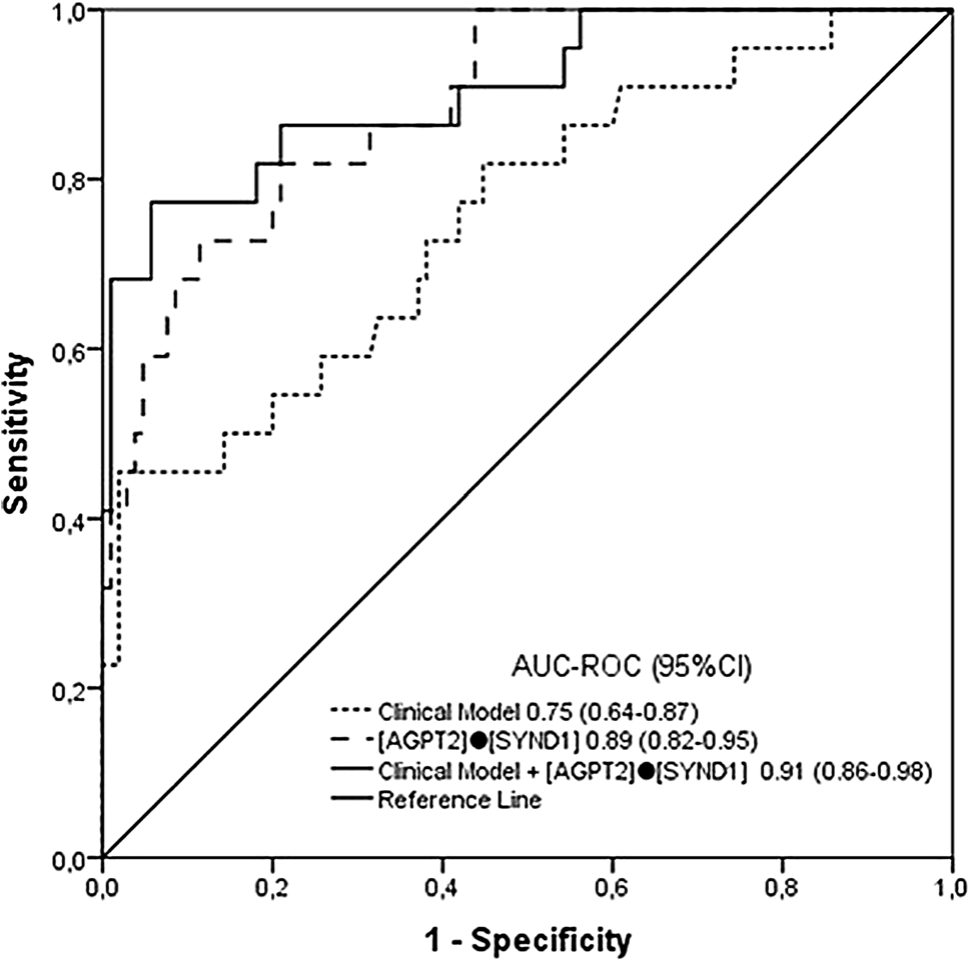
Performance of the clinical model alone, [AGPT2]●[SYND1] and the clinical model + [AGPT2]●[SYND1] for predicting kidney support therapy within the next 72 h. *p* value < 0.001 for all AUC-ROC curves.

The area under the curve (AUC-ROC) values for AGPT2 and syndecan-1 expression and for the clinical model for predicting KST score within 72 h are shown in Fig. 2. Although all the models performed well in predicting KST scores, AGPT2 and syndecan-1 had better performance than did the clinical model. Because these two endothelium-related biomarkers were independently associated with the need for KST according to multivariate analysis even when the other biomarker was added (supplementary Table S2), we hypothesized that they have additive predictive value when used together. According to another biomarker panel study^22^, we applied simple multiplication of two markers ([AGPT2]·[SYND1]), and this combination had an AUC-ROC of 0.89 (95% CI 0.82–0.96), which was better than that of each biomarker alone (Fig. 3).

### The ability of [AGPT2]·[SYND1] to predict KST above clinical prediction criteria

**Figure 4:**
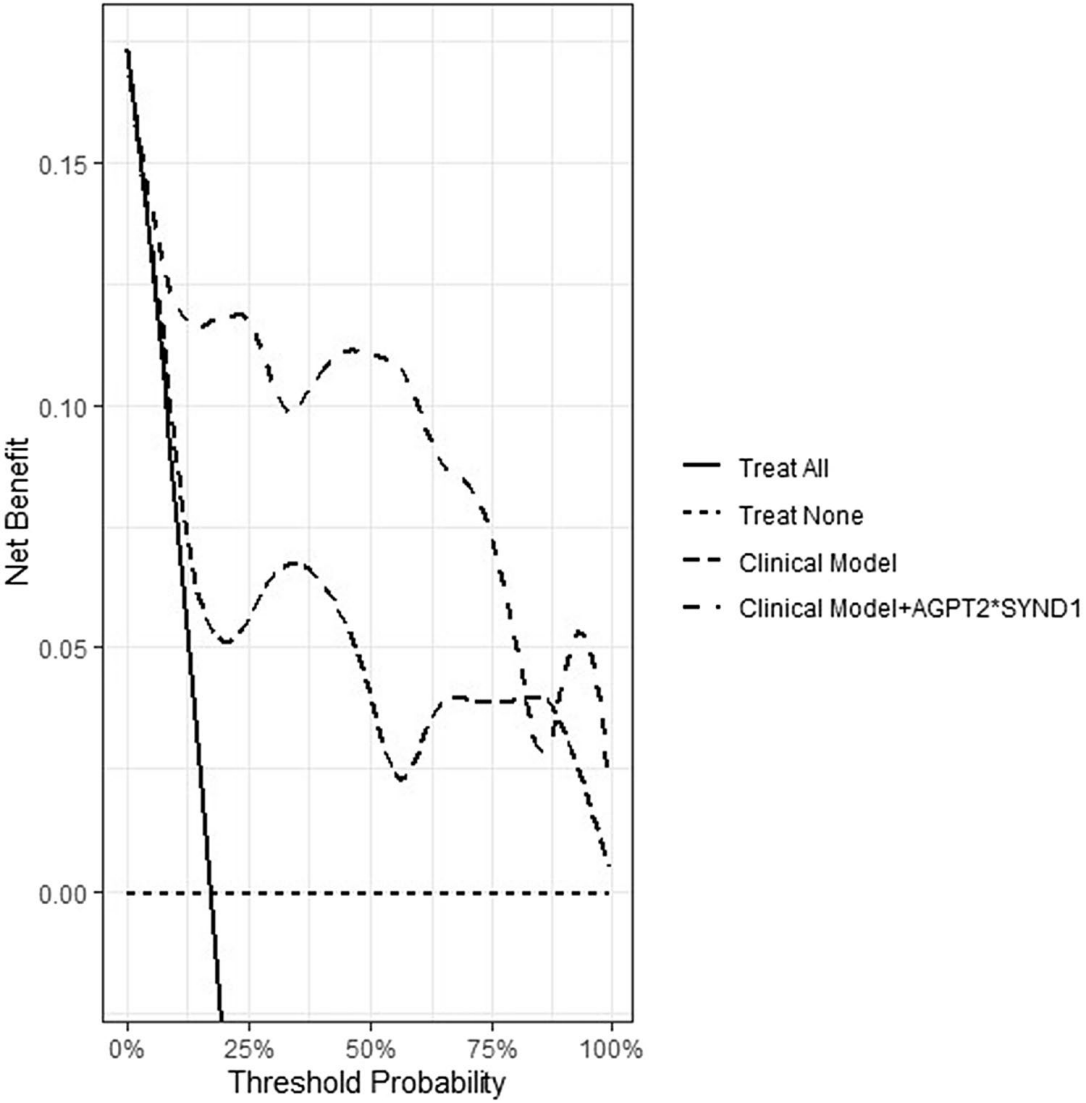
Decision curve analysis demonstrating the clinical utility of the clinical model for predicting kidney support therapy efficacy within 72 h and for enhancing the efficacy of adding [AGPT2]·[SYND1].

The clinical prediction model with clinical variables for the need for KST had an AUC-ROC of 0.75. Adding [AGPT2]·[SYND1] to the clinical model improved the discriminatory ability of the model by 0.91 (95% CI 0.86–0.98, *p* = 0.02 for AUC-ROC comparison against the clinical model alone) (Fig. 3). [AGPT2]·[SYND1] also improved the classification accuracy of KST. The continuous NRI resulting from [AGPT2]·[SYND1] inclusion in the clinical model was amplified by both reclassification of nonevents (i.e., patients without KST) and events (i.e., patients with KST). The continuous NRI was 1.17 (95% CI 0.77–1.56) (Supplementary Fig. S2). The IDI was 0.30 (95% CI 0.15–0.45). A DCA was performed to evaluate the prediction models by calculating the net clinical benefit. In the DCA curves, the x-axis represents the threshold probability, whereas the y-axis represents the net benefit. The DCA curves (Fig. 4) revealed that the clinical model alone was useful, and the addition of [AGPT2]·[SYND1] enhanced the efficacy of the model for predicting KST in almost the threshold probability range, except when it was above 85, indicating its clinical effectiveness.

### Other outcomes

During the 96 h after study inclusion, 28 (22.0%) patients needed KST*.* As expected, the discriminatory capacity of endothelium-related biomarkers and the clinical model were lower than that of KST after 72 h; however, [AGPT2]·[SYND1] and syndecan-1 retained good discriminatory capacity. The values are shown in Supplementary Fig. S3. The ICU mortality rate was 41.7%, and syndecan-1 had the best performance, with an AUC-ROC of 0.72 (95% CI 0.62–0.80) (see supplementary Fig. S4).

## Discussion

In the present study, we evaluated a specific cohort of patients who achieved AKI stage 2 only by the sCr criterion and evaluated several endothelium-related biomarkers as predictors of KST need in the next 72 h. Syndecan-1, a biomarker of endothelial glycocalyx damage, and AGPT2, which antagonize the actions of angiopoietin-1 in the stabilization of the vascular endothelium, were associated with the need for KST. Both biomarkers had good discrimination capacity; moreover, they were independently associated with KST, and a combination of both had better discrimination than each one alone and improved the discrimination capacity of the constructed clinical model.

Although AGPT2 and syndecan-1 are not biomarkers specific to kidney structure, both are related to AKI development under several conditions^23–26^, endothelial injury has an important role in AKI pathophysiology, and both biomarkers can be released from the glomerular endothelium when the kidneys suffer inflammatory injury^27^. While numerous urine biomarkers have been correlated with the onset of AKI^28^ and a specific study has shown an association between urinary syndecan-1 and AKI in a pediatric cohort^29^, most investigations focusing on endothelium-related biomarkers associated with AKI-related outcomes have utilized serum rather than urine^30,31^. Hence, we opted to adhere to this approach.

To the best of our knowledge, our study is the first to evaluate the ability of biomarkers to predict KST in a cohort of patients originally diagnosed with nonoliguric AKI. Additionally, this study is the first to evaluate endothelium-related biomarkers in relation to AKI progression. Interestingly, two endothelium-related biomarkers independently associated with KST (AGPT2 and syndecan-1) were associated with fluid overload in a previous study^31^, one major parameter for initiating KST. In our study, we excluded patients with reduced urine output (< 0.5 ml/kg/h for at least 12 h) and patients with comparable cumulative fluid balance, suggesting that fluid overload is not a confounding variable. Despite not finding a significant association between VCAM-1 or angiopoietin-1 and KST, another study with a specific population of Bothrops envenomation patients demonstrated an association between these biomarkers and mild AKI^32^. However, further studies are necessary to ascertain whether these biomarkers may prove useful in the setting of AKI.

In the last decade, several studies have evaluated whether early KST initiation can ameliorate survival in critically ill patients^33^. All but one of these trials^7^ demonstrated that the timing of KST initiation based on AKI stage, including changes in the serum creatinine concentration with or without urine output, has no advantage for survival or renal recovery. One major point of such trials is that a watch-and-wait strategy can prevent KRT in approximately 40–50% of included patients^6,9,10^. However, such a strategy can lead to AKI-related complications that are associated with adverse outcomes in critically ill patients^5^. In the largest of these trials, although waiting for dialysis was associated with a lower percentage of KST, the patients had a longer duration of KST and a prolonged intensive care unit length of stay^10^.

Postpone KST, whenever possible, may lead to a substantial reduction in the percentage of patients who will ultimately receive KST. However, there is a need to better understand why some patients could avoid KST. In our study, we included only patients with maintained urine output at study inclusion, and although approximately one-third of the patients progressed to AKI stage 2/3 by urine output after 24 h, we cannot assume that our results are valid for patients who have AKI according to the urine output criteria. Although this can be a limitation of our study, deciding on KST initiation in AKI patients with preserved urine output can be especially challenging. In one large study, only 3.2% of patients with stage 3 AKI according to the serum creatinine level needed KST, and this number increased to 35% when there was any reduction in urine output^34^. Additionally, patients with maintained urine output composed a minority of the original study that validated the FST, a diuretic challenge test that has emerged as a practical tool for early prediction of KRT need^35^. Additionally, cumulative fluid balance, a major factor for KST initiation, is more common in oliguric patients, reducing controversy surrounding the indication for KST.

To date, the most studied new biomarkers for predicting the need for KST are directly related to kidney structural damage, i.e., NGAL, KIM-1, and TIMP-1^12^. We evaluated endothelium-related biomarkers that, as cited above, can be released from the kidney endothelium but can also be related to the severity of critical illness. For example, syndecan-1 has recently been demonstrated to reflect organ dysfunction in critically ill patients^36^, and AGPT2 is known to be a prognostic biomarker for critically ill patients, mainly those with acute respiratory distress syndrome (ARDS)^23,37^. Therefore, it is possible that such biomarkers can reflect AKI and illness severity, reflecting, at least partly, the mismatch between the demand placed on kidneys and their capacity.

Several studies have evaluated the ability of clinical variables to predict AKI incidence, and machine learning techniques have been applied for this purpose^13,15–17^. To construct our clinical model, we used the variables cited in several studies and the most important variables in the construction of machine learning. Our clinical model had a discriminative capacity comparable to that of other studies, and either AGPT2 or syndecan-1 alone or in combination had a discriminatory capacity better than that of the constructed clinical model alone. Importantly, the clinical model and [AGPT]·[SYND1] had additive predictive value together with the best discriminatory capacity.

Our study has several implications for the early identification of patients with AKI who need KST. First, studying the timing of the KST can play a role in determining the best approach. The recent ADQI recommendations on AKI biomarkers^12^ highlighted the need for further research on the dynamic assessment of kidney damage and functional biomarkers correlated with clinical data to define the optimal timing of KST. Second, after further studies and validation, additional clinical practice information can be added to patients with stage 2/3 AKI, and urine output can be maintained when clinicians must individualize therapy with a global assessment of the severity of illness, fluid balance, and AKI severity. Finally, our data demonstrated that biomarkers other than those specific to kidney structural damage can help stratify the risk of KST.

Our study has several limitations. First, it was performed in only one center. Additionally, although the recruited patients had a well-defined profile, the sample size is another limitation. Third, although we generally recommended guidelines to initiate KST, this was ultimately a decision made by the attending physician. Finally, we did not compare endothelium-related biomarkers with other biomarkers reported in the literature that predict the need for KST in the AKI setting^38^.

In conclusion, AGPT2 and syndecan-1 demonstrated predictive value for the need for KST in critically ill patients with stage 2 AKI. The combination of AGPT2 and syndecan-1 alone enhanced the predictive capacity of predicting KST beyond clinical variables alone. These findings may contribute to the early identification of patients who will benefit from KST and aid in the management of AKI in critically ill patients.

### Supplementary Information

Supplementary Information.

## Data Availability

The data that support the findings of this study are available from the corresponding author upon reasonable request.
