# Supplementary material for: Endothelium-related biomarkers enhanced prediction of kidney support therapy in critically ill patients with non-oliguric acute kidney injury
Source: Sci Rep. 2024 Feb 21;14:4280. doi: 10.1038/s41598-024-54926-9 (PMC10881963; doi:10.1038/s41598-024-54926-9)
Supplement: Supplementary file 1 — Supplementary Information. [file 41598_2024_54926_MOESM1_ESM.pdf]

Endothelium-related Biomarkers Enhanced Prediction of Kidney Support Therapy in Critically Ill Patients with Non-Oliguric Acute Kidney Injury.

Authors:

Francisco Thiago Santos Salmito, **Sandra Mara Brasileira Mota, Francisco Márcio Tavares Holanda**, **Leticia Libório Santos, Luana Andrade**, **Gdayllon Cavalcante Meneses**, **Nicole Coelho Lopes**, **Leticia Machado de Araújo**, **Alice Maria Costa Martins**, **Alexandre Braga Libório**

Supplementary Figure S1: Forest plots demonstrating that endothelial-related biomarker levels are associated with kidney support therapy initiation 72 hours after study inclusion. Adjusted for baseline sCr, change in sCr after the 24-h previous study and need for vasoactive drugs.

Supplementary figure S2: Net reclassification improvement. Patients who received kidney support therapy (red) above the reference line and those without kidney support therapy (black) under the reference line were better reclassified.

Supplementary figure 3: Diagnostic performance of angiopoietin-2, sydecan-1, and [AGPT2]●[SYND1] and a clinical model including serum creatinine in the last 24 h before study inclusion and the use of vasoactive drugs for kidney support therapy in the next 96 h.

Supplementary figure 4: Diagnostic performance of angiopoietin-2, sydecan-1 and [AGPT2]●[SYND1] for ICU mortality.

Supplementary Figure S1

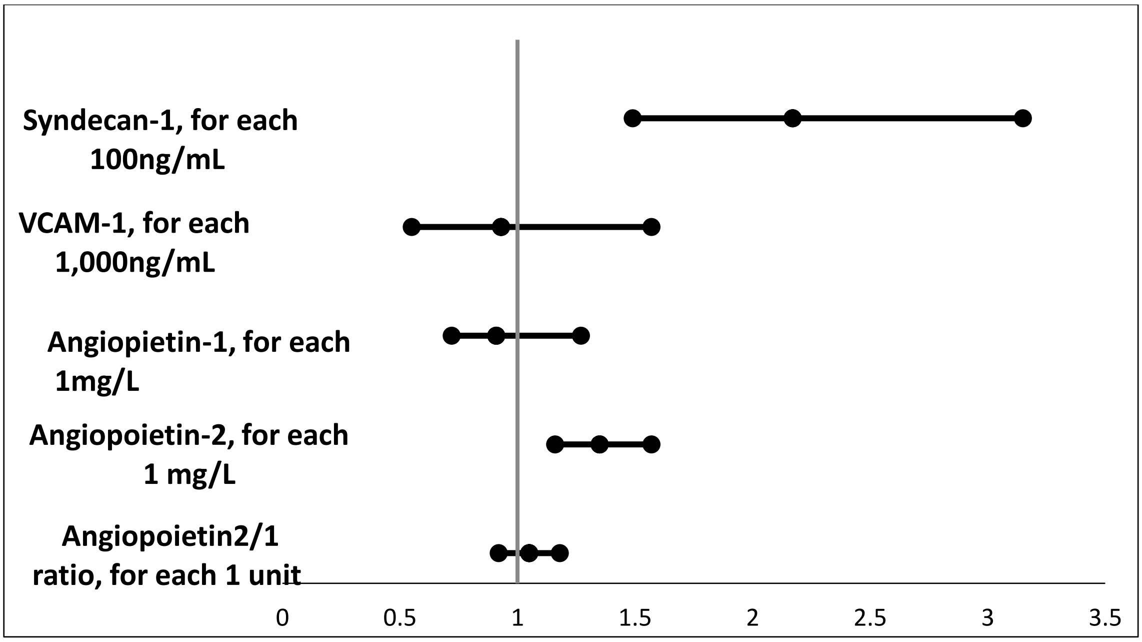

Supplementary figure S2

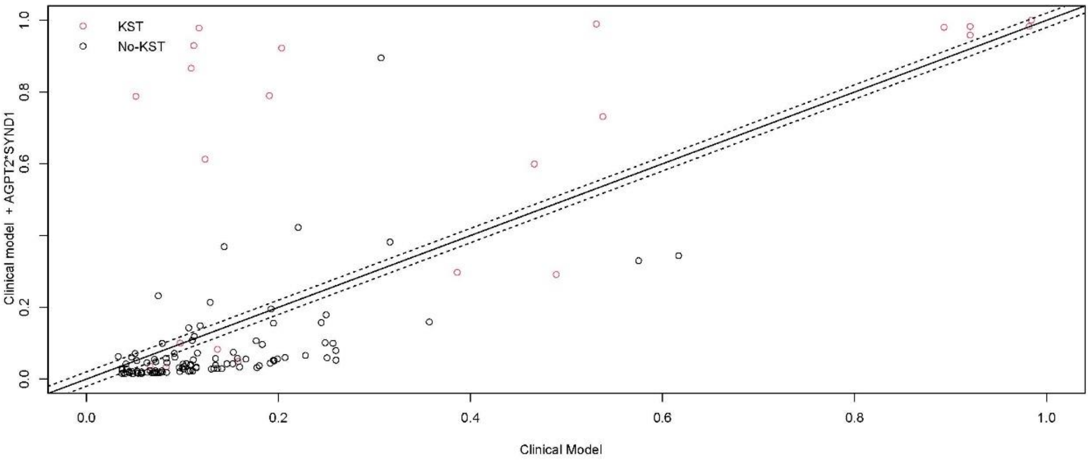

Supplementary figure 3

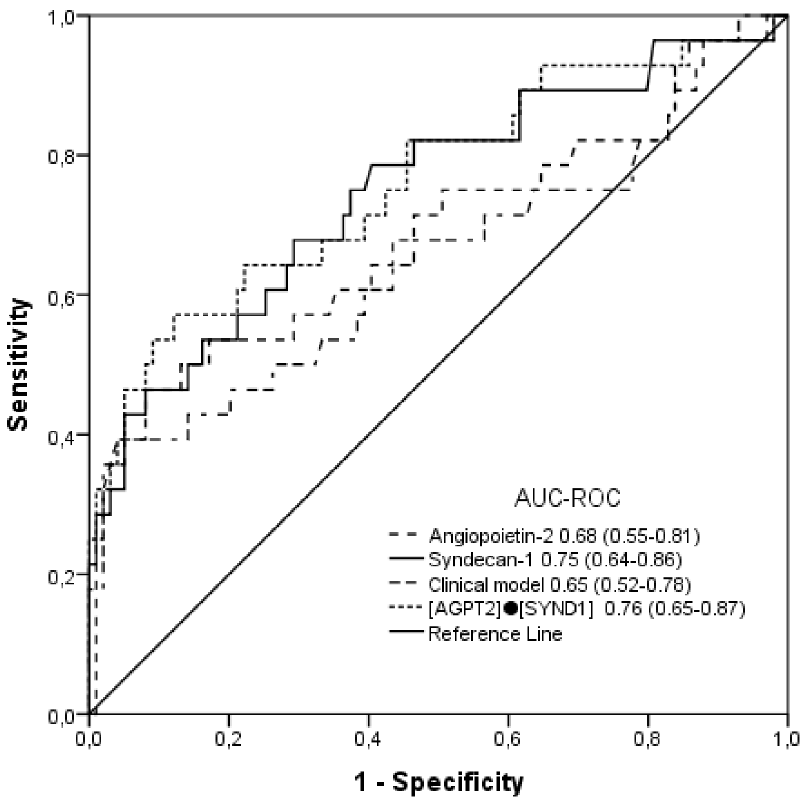

Supplementary figure 4

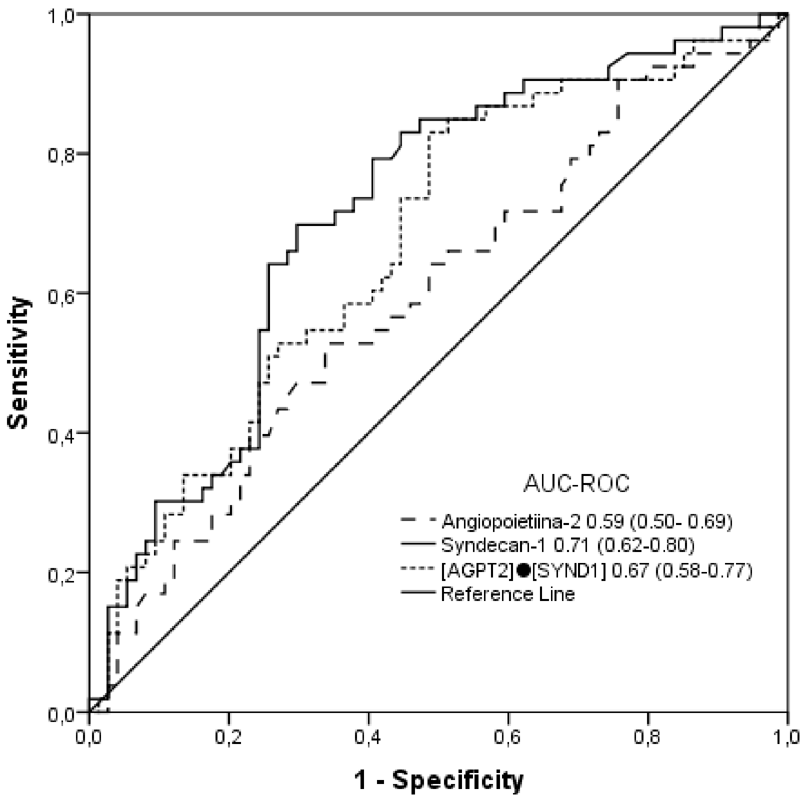

Supplementary table S1: Indications to initiate kidney support therapy according to assistant physician.

Patient number	Hypervolemia	Negative furosemide stress test	Hyperkalemia	Metabolic acidosis	High blood urea	Other
1	●	●				
2		●				
3		●	●			
4				●		
5		●				
6	●			●		
7		●	●			
8		●	●	●		
9						●
10	●	●				
11	●					●
12		●				
13				●	●	
14	●	●				
15	●	●				
16	●	●				
17		●		●		
18	●	●				
19			●	●	●	
20	●					
21	●			●		
22			●			●

Supplementary Table S2: Endothelial-related biomarker levels associated with kidney support therapy initiation 72 hours after study inclusion after adjusting for baseline sCr, change in sCr in the 24 h previous study inclusion and need for vasoactive drugs. Both biomarkers were maintained in the same model for adjustment.

	**Adjusted OR (95%CI)**	**P**
Syndecan-1, for each 100ng/mL	1.93 (1.33-2.82)	0.001
Angiopoietin-2, for each 1 mg/L	1.26 (1.07-1.52)	0.009
